# A Novel Blended Transdiagnostic Intervention (eOrygen) for Youth Psychosis and Borderline Personality Disorder: Uncontrolled Single-Group Pilot Study

**DOI:** 10.2196/49217

**Published:** 2024-04-01

**Authors:** Shaunagh O'Sullivan, Carla McEnery, Daniela Cagliarini, Jordan D X Hinton, Lee Valentine, Jennifer Nicholas, Nicola A Chen, Emily Castagnini, Jacqueline Lester, Esta Kanellopoulos, Simon D'Alfonso, John F Gleeson, Mario Alvarez-Jimenez

**Affiliations:** 1 Orygen Parkville Australia; 2 Centre for Youth Mental Health University of Melbourne Melbourne Australia; 3 Health Brain and Mind Research Centre School of Behavioural and Health Sciences Australian Catholic University Melbourne Australia; 4 School of Computing and Information Systems University of Melbourne Melbourne Australia

**Keywords:** digital intervention, blended care, youth mental health, transdiagnostic intervention, psychotic disorders, borderline personality disorder, digital health, mobile phone

## Abstract

**Background:**

Integrating innovative digital mental health interventions within specialist services is a promising strategy to address the shortcomings of both face-to-face and web-based mental health services. However, despite young people’s preferences and calls for integration of these services, current mental health services rarely offer blended models of care.

**Objective:**

This pilot study tested an integrated digital and face-to-face transdiagnostic intervention (eOrygen) as a blended model of care for youth psychosis and borderline personality disorder. The primary aim was to evaluate the feasibility, acceptability, and safety of eOrygen. The secondary aim was to assess pre-post changes in key clinical and psychosocial outcomes. An exploratory aim was to explore the barriers and facilitators identified by young people and clinicians in implementing a blended model of care into practice.

**Methods:**

A total of 33 young people (aged 15-25 years) and 18 clinicians were recruited over 4 months from two youth mental health services in Melbourne, Victoria, Australia: (1) the Early Psychosis Prevention and Intervention Centre, an early intervention service for first-episode psychosis; and (2) the Helping Young People Early Clinic, an early intervention service for borderline personality disorder. The feasibility, acceptability, and safety of eOrygen were evaluated via an uncontrolled single-group study. Repeated measures 2-tailed *t* tests assessed changes in clinical and psychosocial outcomes between before and after the intervention (3 months). Eight semistructured qualitative interviews were conducted with the young people, and 3 focus groups, attended by 15 (83%) of the 18 clinicians, were conducted after the intervention.

**Results:**

eOrygen was found to be feasible, acceptable, and safe. Feasibility was established owing to a low refusal rate of 25% (15/59) and by exceeding our goal of young people recruited to the study per clinician. Acceptability was established because 93% (22/24) of the young people reported that they would recommend eOrygen to others, and safety was established because no adverse events or unlawful entries were recorded and there were no worsening of clinical and social outcome measures. Interviews with the young people identified facilitators to engagement such as peer support and personalized therapy content, as well as barriers such as low motivation, social anxiety, and privacy concerns. The clinician focus groups identified evidence-based content as an implementation facilitator, whereas a lack of familiarity with the platform was identified as a barrier owing to clinicians’ competing priorities, such as concerns related to risk and handling acute presentations, as well as the challenge of being understaffed.

**Conclusions:**

eOrygen as a blended transdiagnostic intervention has the potential to increase therapeutic continuity, engagement, alliance, and intensity. Future research will need to establish the effectiveness of blended models of care for young people with complex mental health conditions and determine how to optimize the implementation of such models into specialized services.

## Introduction

### Background

The evolution of specialist early intervention services for youth represents a major global reform of mental health services [1-3]. However, there are shortcomings that remain to be addressed for these services to fully deliver on their promise; for example, 42% of young people drop out of treatment by the third therapy session [4], indicating low engagement rates with early intervention services [5]. Furthermore, those who continue treatment receive time-limited support [6], and up to 80% of young people with severe mental health conditions will incur repeated relapses, leading to long-term disability and high societal cost [7,8]. Estimates suggest that the cost associated with recurring mental ill-health is up to 5 times that of nonrelapsing presentations [9]. Even when young people receive evidence-based treatment, its effectiveness is limited [10]; for example, between one-third and two-thirds of young people do not experience symptom reduction [11], and functional impairment often remains an issue after remission [12].

Digital technologies have the potential to address these challenges and limitations by enhancing the accessibility, impact, reach, and cost-effectiveness of youth mental health (YMH) services [13,14]. Many young people, recognizing their need for help, are turning to technology, including smartphone apps, websites, and social media, to self-manage their mental well-being [15]. Research findings support the efficacy of digital mental health interventions in improving treatment outcomes in severe mental health conditions such as psychosis [16-18] and borderline personality disorder (BPD) [19]. Web-based treatment programs have also demonstrated efficacy comparable to that of face-to-face psychotherapy [20-22], and self-guided smartphone-based mental health interventions are proving to be promising self-management tools for depression and anxiety symptoms [23].

However, despite the evidence for the effectiveness of digital interventions, limitations have also been reported, such as high attrition rates [24,25], a focus on mild to moderate mental health conditions [26-28], a focus on single disorders ignoring potential comorbidity [29], and a lack of integration within clinical settings [30,31]. Factors affecting attrition and dropout from digital interventions have been identified, such as a lack of personalization within digital interventions and severe mental disorders hampering engagement with interventions [32]. Most of the first generation of digital interventions have also been deployed and evaluated without face-to-face care, generating a divide between face-to-face and digital supports [33]. Furthermore, clinical trials of digital interventions often recruit highly motivated early adopters from the community, resulting in poor generalizability of the findings in clinical settings [33,34].

Blended care refers to treatment that includes face-to-face and digital elements, both of which contribute to the treatment process and can be integrated or offered sequentially [35,36]. Blended models of care offer an innovative approach to address the limitations of both face-to-face and digital therapy for young people with serious mental illness, while maintaining the strengths of both modalities [36,37]. This integrated approach is in line with World Health Organization recommendations [38] and young people’s preferences, as well as national and international calls for the integration of web-based and in-person mental health services [39]. Young people have indicated that blended models of care could enhance clinical care by increasing accessibility and continuity of care, providing access to posttherapy support, and strengthening the relationship with their clinician [40]. However, despite young people’s preferences and calls for the integration of face-to-face and web-based services [16,41], current mental health services rarely provide this type of integrated web-based support [42]. Barriers to implementing digital interventions in clinical settings have also been identified, such as a lack of time for clinicians and skepticism toward digital interventions [43], and these need to be taken into account when developing blended interventions. Furthermore, there is limited research testing blended models of care as a treatment approach [37,44], despite the demonstrated efficacy of stand-alone digital interventions [45-47].

Furthermore, transdiagnostic interventions that target underlying mechanisms or symptoms that are common across multiple mental disorders have the potential to provide more effective, personalized, and engaging treatment, addressing comorbidity and being applicable to a wider range of young people [48,49]. Evidence also suggests that transdiagnostic interventions may be at least as effective, more engaging, and easier to scale up in real-world clinical settings compared with single-disorder interventions [50,51].

As blended transdiagnostic interventions for first-episode psychosis and BPD have not yet been evaluated, a 3-month pilot evaluation of a blended transdiagnostic digital intervention (eOrygen) designed to enhance the accessibility, responsiveness, and impact of face-to-face specialized YMH clinical services for youth psychosis and BPD was carried out. Pilot studies are an important first step before running a full-powered clinical trial because they focus on whether an intervention can be carried out, whether it would be worth proceeding with it, and how to proceed before focusing on evaluating the effectiveness of the intervention [52].

### Objectives

The primary objective of the eOrygen pilot was to evaluate the feasibility, acceptability, and safety of an integrated web-based clinic that blends moderated online social therapy (MOST) support with face-to-face specialized YMH clinical services for youth psychosis and BPD. A secondary aim of the pilot was to assess changes in key clinical and psychosocial outcomes for young people from the point of enrollment in eOrygen to after the intervention. Furthermore, the failure to integrate digital technologies into routine practice is well documented [53], and, therefore, an additional objective of this study was to understand young people’s and clinicians’ experiences of barriers and facilitators to using a blended model of care in clinical practice.

## Methods

### Study Design

This was a 3-month multisite pre-post single-group pilot study conducted at the Early Psychosis Prevention and Intervention Centre (EPPIC) and the Helping Young People Early (HYPE) Clinic at Orygen Youth Health in Melbourne, Victoria, Australia. EPPIC provides services for young people aged 15 to 25 years experiencing their first episode of psychosis, and admission to the service is based on a clinical assessment determining the presence of full-threshold first-episode psychosis, including full-threshold psychotic symptoms such as hallucinations, delusions, or formal thought disorder [54]. The HYPE Clinic offers an early intervention program for young people aged 15 to 25 years with BPD, and intake to the service is based on meeting ≥3 BPD criteria according to the *DSM-5* (*Diagnostic and Statistical Manual of Mental Disorders* [Fifth Edition]) Text Revision [55]. These services deliver specialized early interventions, with treatment offered from 6 months to a maximum of 2 years. Each young person at Orygen Youth Health receives case management by a dedicated mental health clinician, with additional assessment and treatment support provided by a psychiatrist. Each year, approximately 155 young people access HYPE Clinic services, and approximately 250 young people access EPPIC services.

### Sample Size

It is recommended that the sample size of a pilot study be approximately 10% of the sample size projected for the larger parent study [56]. At present, there is little definitive research available to determine the sample size of the larger parent randomized controlled trial for a transdiagnostic blended model of care, hence the importance of conducting this pilot feasibility study. In a recent study using the same technology, a total sample of 140 participants was determined to detect changes in social functioning at 90% power, accounting for attrition of 20% [57]. Given this, we proposed to recruit 25 clinicians and 1 to 2 young people per clinician, resulting in an anticipated 25 to 50 young people in the study.

### Ethics Approval

Ethics approval was obtained from the Melbourne Health Human Research Ethics Committee (HREC/49492/MH-2019).

### Participants and Procedure

The participants were 18 mental health clinicians and 33 young people recruited by participating clinicians and the Orygen research team across HYPE Clinic and EPPIC clinical services. Clinician recruitment took place over a 2-month period, and the recruitment of young people took place over a 4-month period from May 15 to September 25, 2020.

#### Young People

##### Inclusion and Exclusion Criteria

Young people were recruited via their clinician and the research team. Clinicians within each service were invited to identify potentially eligible young people based on the following criteria: (1) aged 15 to 25 years (inclusive), (2) currently receiving treatment at the HYPE Clinic or EPPIC, (3) engaged with treatment as judged by the treating clinician and not approaching discharge from service, (4) willing to nominate an emergency contact person, (5) have regular and ongoing internet and telephone access, and (6) able to give informed consent and comply with study procedures. The exclusion criteria were as follows: (1) young people with an intellectual disability who were unable to meet the cognitive demands of the web-based intervention, interfering with the likelihood of benefiting from the intervention as judged by their treating clinician; and (2) young people with an inability to converse in, or read, English. There were no specific exclusion criteria related to level of suicide risk or interpersonal hostility (ie, a consideration for harm to self or others while engaging within a web-based social network). However, clinicians were consulted on a case-by-case basis regarding participant suitability, and clinician judgment regarding suitability could be reassessed at any time. The exclusion criteria for the pilot study were kept to a minimum both to facilitate the recruitment process and to ensure that the intervention was tested and adequately mirrored the real-world characteristics of the broad population of young people accessing specialist YMH services. Furthermore, to mirror the intended real-world implementation of eOrygen, these exclusion criteria were assessed and monitored by the participating clinicians.

##### Recruitment Process

Once eligibility was determined, eligible young people were invited to participate in the study by the research team. Among the HYPE Clinic clients, of the 106 young people who were assessed for eligibility for this study, 42 (39.6%) met the inclusion criteria; however, 23 (55%) of these 42 young people could not be approached because of their involvement with another research study. Thus, 19 young people were approached to participate, and 15 (79%) agreed to participate and enrolled in the study, whereas 4 (21%) declined. Of the 106 young people assessed for eligibility, 64 (60.4%) were ineligible to participate in this study owing to clinical risk, poor engagement with treatment, a lack of access to technology, age, or because they were approaching discharge.

Among the EPPIC clients, of the 59 young people who were assessed for eligibility, 43 (73%) met the inclusion criteria; however, 3 (7%) of these 43 young people were already participating in another research study. Thus, 40 young people were approached to participate, and 20 (50%) agreed to participate, whereas 11 (28%) declined, and 9 (23%) could not be contacted by the research team. Of the 20 young people who agreed to participate, 2 (10%) could not be contacted to complete their baseline assessments and did not enroll in the study; the remaining 18 (90%) enrolled in the study. However, of these 18 young people, 7 (39%) were lost to follow-up during the study period (n=3, 43% before onboarding to the eOrygen platform and n=4, 57% before completing the 3-month follow-up assessments). The young people lost to follow-up were unresponsive to telephone calls and messages from the research team but were still engaged with face-to-face treatment with their clinicians. Of the 59 young people assessed for eligibility, 16 (27%) were ineligible to participate in this study for the same aforementioned reasons.

Participant consent was obtained from those interested in participating and parental or guardian consent was also obtained for young people aged <18 years.

##### Assessments

The consenting young people were contacted at baseline via email and telephone to complete baseline measures before setting up their eOrygen user account. The young people then continued treatment with their clinician while having access to the eOrygen platform for 3 months. They were contacted again at the end of the 3-month intervention to complete the postintervention assessments.

During postintervention follow-up telephone call assessments, the young people were asked whether they were willing to be contacted for a subsequent qualitative interview, and 19 (58%) of the 33 young people agreed to be followed up. After the intervention phase, a randomly selected subgroup comprising 12 (63%) of the 19 young people who agreed to be contacted were invited via SMS text messaging to participate. These semistructured qualitative interviews were designed to explore their experiences with the eOrygen platform. The primary goal of these interviews was to identify both barriers and facilitators to their engagement with the intervention.

Of the 12 young people approached after the intervention, 1 (8%) declined to participate (no reason provided), and 2 (17%) agreed to participate but did not attend the scheduled interviews and were not able to be contacted; thus, 9 (75%) participants successfully completed the interview process. However, a technical issue resulted in a recording failure during 1 (11%) of the 9 interviews, and it could not be included in the subsequent analysis.

The interviews were all conducted via Zoom (Zoom Video Communications, Inc), and interview times ranged from 22 to 38 minutes. Participants were recruited and interviewed by a study research assistant and author EC. Interview questions were underpinned by a user-centered design approach [24] and focused on the following aspects: what initially interested participants about using the eOrygen platform; the experience of onboarding; hopes and expectations; barriers and facilitators; and the overall experience of the therapy journeys, clinical and peer support, and community features of the platform.

#### Clinicians

All mental health clinicians employed at the HYPE Clinic and EPPIC were eligible for inclusion in this study. Clinicians attended a workshop focused on learning about the background of the intervention, including previous empirical findings using the same technology and how to use the eOrygen platform. The latter aspect concerned how to use the intervention functions and set up an account, with suggestions provided on how to integrate this into the clinicians’ work with young people, with the possibility of using it within and between face-to-face sessions as they felt appropriate. This included clinical case studies that applied to the populations of both HYPE Clinic and EPPIC services and were coauthored by clinicians at these services. Clinicians were also provided a training manual that was used to help them navigate the platform during the workshop and also to keep and reuse as necessary when navigating the intervention platform independently. As the workshop was held before recruiting young people to the study, clinicians were provided with a training video at a later date describing once again how to use the intervention platform features.

Eligible clinicians were then invited to identify eligible young people who met the aforementioned inclusion criteria. All participating clinicians had at least 1 young person using the eOrygen platform. The clinicians were contacted via email at baseline to complete the clinician-rated measures. They were then invited to use the eOrygen platform with their clients for 3 months. At the end of the intervention, they were contacted again via email to complete the postintervention clinician-rated measures.

After the postintervention phase, an invitation was extended to all 18 clinicians to participate in a structured focus group session. The aim of this session was to delve into the obstructive and conducive factors influencing the effective implementation of the eOrygen platform into routine clinical practice. This evaluative process was grounded in the Consolidated Framework for Implementation Research (CFIR), a recognized and widely used framework for assessing the determinants influencing implementation in health care settings [58]. The interview schedule was designed by author LV and based on the CFIR constructs that were identified via both formal and informal consultation with the specialist service settings throughout the intervention period. Among the notable constructs under consideration were those related to evidence, adaptability, complexity, needs and resources, and self-efficacy.

Clinicians were invited via email and supported by their line managers to attend. Of the 18 clinicians, 15 (83%) were available to participate in the focus groups that were conducted over Zoom. Because of the number of clinicians available to participate, the focus groups were divided into 3 distinct sessions to facilitate more extensive discussions and allow individual clinicians ample opportunity to share their insights and experiences. Authors LV, DC, and EK each conducted 1 of the 3 parallel sessions.

### Intervention: eOrygen

The eOrygen intervention was based on Orygen Digital’s MOST model, which was the first digital solution to offer continuous integrated face-to-face and digital care to young people across the mental health diagnostic and severity spectrum and stages of treatment [59-61]. In partnership with young people, the MOST model was iteratively developed by a multidisciplinary team of researchers, clinical psychologists, programmers, creative writers, graphic artists, and experts in human-computer interaction [61-63]. A recent clinical trial with young people with psychosis demonstrated that an intervention based on the MOST model was effective in improving vocational and educational outcomes as well as reducing the use of emergency services; in addition, it was cost-effective, with evidence of a dose-response effect [60,64,65].

**Figure 1:**
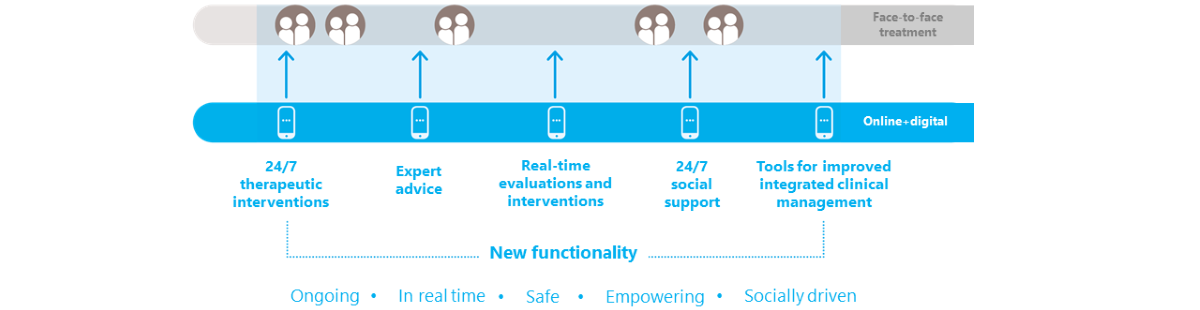
eOrygen as a blended model of care.

The eOrygen intervention was a purpose-built web-based platform designed to integrate face-to-face and web-based support for young people experiencing mental ill-health (Figure 1). This was achieved through the use of both clinician and young person user accounts, making it possible for young people and their treating clinician to use the platform throughout the treatment process, during their face-to-face sessions or between sessions. Young people could also use the platform in a self-directed way, but no automated prompts or reminders were provided for young people to use eOrygen between sessions, unless clinicians made suggestions to their young clients using the platform.

The platform was designed to enhance, not replace, recommended treatments for mental health conditions (eg, clinician-administered cognitive behavioral therapy). eOrygen comprised interactive user-directed psychosocial interventions (*therapy journeys*), a social network, clinical moderation, and peer support.

**Figure 2:**
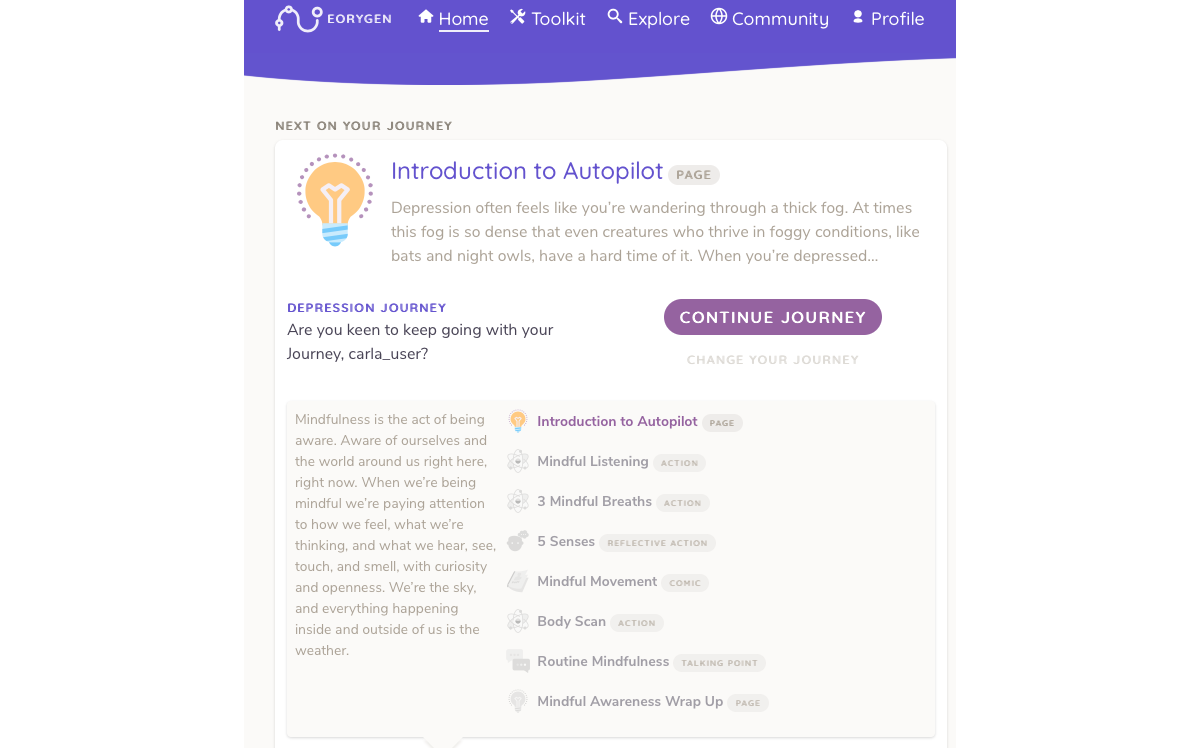
eOrygen therapy journey.

*Therapy journeys* comprised collections of *therapy activities* relating to different themes. Themes related to the treatment of mental ill-health, such as managing social anxiety, anxiety, and depression, as well as social functioning. Users were assigned a suggested *therapy journey* based on their responses to a questionnaire they completed after being onboarded to the eOrygen platform, providing personalized content specific to their individual mental health concerns (Figure 2). Users could complete multiple *therapy journeys*, and clinicians or young people could change the assigned journey.

*Therapy activities* could be accessed as part of a *therapy journey* or as stand-alone activities via the *explore* function. The *explore* function enabled young people to use a search bar to locate therapy content of interest, and eOrygen clinicians could also recommend personalized content to young people using this function. *Therapy activities* included *activities*, *comics*, *talking points*, and *actions*. *Activities* comprised written content, and *comics* comprised storyboard panels focusing on a particular therapeutic theme and target related to the treatment of mental ill-health challenges. *Talking points* enabled participants to propose a solution to identified problems (eg, how to incorporate mindfulness into everyday activities), which encouraged social problem-solving and effective peer modeling. *Actions* were behavioral prompts that young people could complete to translate learning on a mental health topic into behavior change.

**Figure 3:**
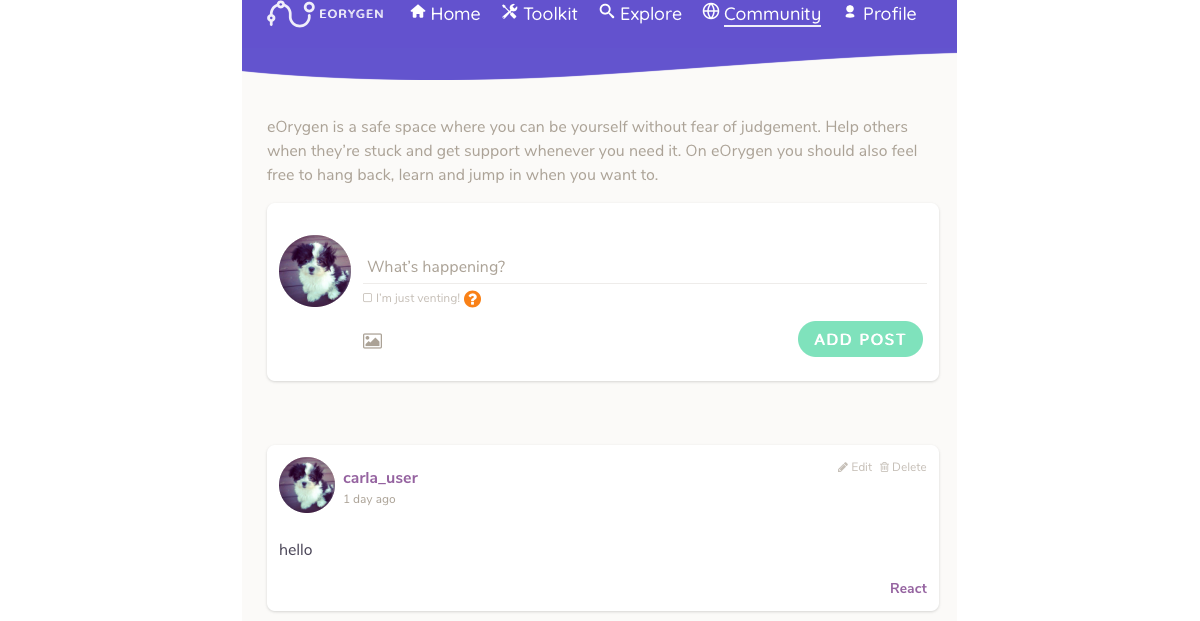
eOrygen social network.

The eOrygen *social network* was moderated by trained peer workers, who were young people who had a lived experience of mental illness. The *social network* included a community newsfeed and individual profile pages where participants and peer workers could create *posts* to share thoughts, information, pictures, and videos (Figure 3). They could also respond to other users’ posts through *comments* or *reactions*. *Reactions* were designed to facilitate social support (eg, “I get you” and “Thinking of you”). Likewise, *talking points* were designed as collaborative spaces to discuss specific topics by leaving *comments*. Young people were also able to receive direct support from peer workers and clinicians on the platform via private *messages*.

### Outcome Measures

#### Feasibility

The feasibility of eOrygen was measured by tracking recruitment to the study, which is in line with other studies testing the feasibility of digital interventions [66]. Although no a priori minimum or maximum number of participants per clinician or per clinic was specified, to accurately assess feasibility, we anticipated recruiting approximately 15 clinicians from EPPIC and 1 to 2 young people per each participating clinician as well as 10 clinicians from the HYPE Clinic and 1 to 2 young people per clinician.

Feasibility was indicated if (1) the recruitment goal was met and (2) the participant refusal rate was <50%. If the recruitment goal was not met after 2 months of recruitment, barriers to recruitment were to be identified.

#### Acceptability

Intervention acceptability was measured via responses to a feedback questionnaire. The pilot was considered to indicate acceptability of the eOrygen intervention if at least 90% of the young people reported that they would recommend it to others, which is in line with a previous pilot study testing the acceptability of a digital mental health intervention [59].

#### Safety

Intervention safety was measured by analyzing reports of any adverse events, tracking the security of the web-based system, and analyzing responses to a feedback questionnaire, following a similar protocol for a previous study [7,67]. An adverse event was defined as any unfavorable or unintended sign, symptom, or disease temporally associated with the use of the intervention, whether or not it was related to the intervention. A serious adverse event was defined as any untoward medical occurrence that could be life threatening, result in death, require inpatient hospitalization, or result in persistent or significant disability. All participants were closely monitored by clinical moderators for adverse events and serious adverse events. The research team members were trained in study procedures, including adverse event assessments, and attended good clinical practice training. In addition, treating clinicians were asked to report any adverse events, including suicide attempts and serious self-harm, to the research team.

The pilot was considered to indicate the safety of eOrygen if (1) there were no unlawful entries recorded in the eOrygen system during the pilot, (2) no young people experienced a serious adverse event as a result of their engagement with the system during the 3-month intervention period, (3) at least 95% of the young people reported it to be safe via the feedback questionnaire, and (4) clinical and social measures did not show a worsening pattern over the course of the study.

Safety was also reported by assessing pre-post changes in BPD symptomatology for HYPE Clinic participants as measured by the 23-item version of the Borderline Symptom List [68], and pre-post changes in psychotic symptoms for EPPIC participants as measured by 3 items of the expanded Brief Psychiatric Rating Scale (version 4.0), including suspiciousness, hallucinations, and unusual thought content [69].

#### Potential Clinical Effects

Potential clinical effects were assessed by measuring pre- to postintervention changes in clinical and psychosocial outcomes at baseline and at 3 months. Clinician-rated measures included social and occupational functioning as measured by the Social and Occupational Functioning Assessment Scale [70] and therapeutic alliance (TA) as measured by the Working Alliance Inventory–Short Revised (therapist version) [71].

Young people self-report measures included depression as measured by the Patient Health Questionnaire-9 [72]; TA with their face-to-face clinician as measured by the Working Alliance Inventory–Short Revised (client version) [73]; psychological well-being as measured by the Flourishing Scale [74]; self-determination as measured by the Basic Psychological Needs Satisfaction Questionnaire [75]; loneliness as measured by the University of California, Los Angeles Loneliness Scale (version 3) [76]; social isolation as measured by the Friendship Scale [77]; social anxiety as measured by the Social Interaction Anxiety Scale [78]; stress as measured by the Perceived Stress Scale [79]; and psychological distress as measured by the 10-item Kessler Psychological Distress Scale [80]. These measures have been validated in a youth population and were chosen for their demonstrated reliability. All baseline measures were completed before onboarding participants to the eOrygen platform, and all assessments were completed via Qualtrics (Qualtrics International Inc) where possible or otherwise administered by a research assistant over the telephone.

#### Satisfaction Survey Feedback

Purpose-designed questionnaires administered via Qualtrics were used to assess user satisfaction and user feedback.

### Statistical Analyses

#### Overview

This study used a mixed methods design involving both quantitative and qualitative data, which allowed for a more robust analysis [81]. Quantitative data assessing the feasibility, acceptability, safety, and potential clinical effects of the intervention were measured at baseline and at 3-month follow-up. Qualitative data were used to assess young people’s and clinicians’ barriers and facilitators to implementing eOrygen within clinical services, which enhanced our understanding of the feasibility and acceptability aspects of the intervention and provided valuable information for designing a full-powered trial that would have not been achieved through quantitative data strands alone.

#### Quantitative Analyses

Chi-square tests showed no differences between the clinical sites in baseline demographic and clinical and psychosocial outcomes. Therefore, data were pooled, repeated measures 2-tailed *t* tests were conducted, and within-group effect sizes (Cohen *d*) were reported for changes in pre- to postintervention scores on effectiveness outcome measures. Parametric and nonparametric correlations were conducted to explore the association between the use of eOrygen and the degree of change between before and after the intervention on effectiveness outcome measures, but no relationships between use and effectiveness outcomes were found.

Aggregated data from the user satisfaction questionnaire and descriptive statistics from insights into using eOrygen were reported as exploratory findings. The data for the acceptability criterion of whether young people would recommend eOrygen to others were derived from insights into eOrygen descriptive statistics. The data for 1 of the safety criteria regarding whether young people felt safe using eOrygen were derived from the user satisfaction questionnaire. Statistical analyses were performed using SPSS (version 27.0; IBM Corp).

#### Qualitative Analyses

##### Young People

Given the user-centered design approach, thematic analysis was considered the most appropriate method of data analysis [82]. The data analysis was conducted by author EC under the supervision of author LV.

The analysis process used an inductive approach in which EC explored young people’s experiences for factors relating to barriers and facilitators to young people’s engagement with the blended model of care. To gain familiarity with the data set, the interview transcripts were read and reread. Subsequently, initial codes were applied to the transcripts to identify relevant factors to engagement. Any recurring codes, both within and across different transcripts, were identified and recorded.

Following the principles of thematic analysis, these codes were then grouped into preliminary themes and subjected to thorough review in relation to all other identified themes. Some themes were identified as superordinate, representing broader categories of experience, whereas others assumed subordinate positions, delineating into subthemes.

##### Clinicians

Three focus groups were conducted by authors DC, EK, and LV with 15 service clinicians overall. Focus groups comprised a mix of both HYPE Clinic and EPPIC clinicians and lasted between 31 and 44 minutes. Interview questions were underpinned by the CFIR [58], which is one of the most widely used frameworks for identifying factors impacting implementation outcomes [83]. The CFIR comprises 39 constructs across 5 domains. The domains identified as most relevant to this implementation setting in the preimplementation phase were *inner and outer settings*, *individual characteristics*, and *innovation characteristics*. The clinician focus groups underwent deductive coding by authors JN and LV in accordance with the CFIR. JN and LV then engaged in a rigorous discussion to examine how the attributes within the identified domains acted as either obstacles or enablers to the implementation of eOrygen in this clinical setting.

#### Use Metrics

Use metrics were used to measure engagement with eOrygen. *Number of active days* was used as an overall metric for platform use and referred to the number of days a young person accessed eOrygen after completing onboarding until the end of the 3-month intervention period.

*Therapy views* comprised the number of times a young person opened *therapy activities* via a *therapy journey* or via a search function. Users were encouraged to revisit activities, and repeat views were counted within the number of *therapy views*. *Journey components completed* was a count of the number of unique *therapy activities* a user completed within a *therapy journey*.

Engagement with the social networking component of eOrygen was measured by the *number of posts*, *comments*, and *reactions made* by young people on the social network. This included *posts made* on the newsfeed and on individual users’ profiles. *Comments made* could be in response to *posts made* by other users or peer workers or *talking point* therapy activities. *Reactions made* could be in response to any post or comment on the social network.

There was also a chat function where young people could communicate with eOrygen staff (clinicians and peer workers) through private direct messages on the platform. Engagement with clinicians and peer workers was measured by the *number of messages sent* (by young people) and the *number of messages received* (from clinicians or peer workers).

## Results

### Demographics

Participants were aged between 15 and 24 (mean 19.48, SD 2.84) years, and the majority were female individuals (21/33, 64%). Of the 33 participants, 27 (82%) were born in Australia, 7 (21%) spoke languages other than English at home, 3 (9%) identified as Aboriginal or Torres Strait Islander, 9 (27%) were engaged in paid work, and 22 (66%) were studying part time or full time (Table 1).

**Table 1:** Participants’ descriptive statistics (total and by each clinical site).

			Total sample (n=33)		Clinical sites		
					HYPE^a^ Clinic (n=15)		EPPIC^b^ (n=18)
Age (years), mean (SD)			19.48 (2.84)		18.8 (2.70)		20.21 (2.89)
**Age (years), n (%)**							
	≤18	13 (45)^c^		8 (53)^d^		5 (36)^e^	
	>18	16 (55)^c^		7 (47)^d^		9 (64)^e^	
**Gender, n (%)**							
	Man	7 (21)		3 (20)		4 (22)	
	Woman	21 (64)		11 (73)		10 (56)	
	Transgender, genderqueer or nonconforming, and other (including multiple gender selections)	5 (15)		1 (7)		4 (22)	
**Aboriginal or Torres Strait Islander**							
	Yes	3 (9)		2 (13)		1 (6)	
	No	29 (88)		13 (87)		16 (89)	
	Unsure	1 (3)		0 (0)		1 (6)	
**Currently studying**							
	Yes	22 (67)		10 (67)		12 (67)	
	No	11 (33)		5 (33)		6 (33)	
**Currently employed**							
	Yes	9 (27)		4 (27)		5 (28)	
	No	24 (73)		11 (73)		13 (72)	

^a^HYPE: Helping Young People Early.

^b^EPPIC: Early Psychosis Prevention and Intervention Centre.

^c^n=29.

^d^n=15.

^e^n=14.

### Feasibility

We sought to recruit 25 clinicians; the final number of clinicians enrolled in the study was 18 (78%). The final number of young people recruited was 33 (which exceeded our goal of 1 young person per clinician). We also anticipated that the refusal rate would be <50%, which it was at 25% (15/59).

### Acceptability

We exceeded our acceptability goal, with 92% (22/24) of the young people reporting that they would recommend eOrygen to others (Multimedia Appendix 1).

### Safety

There were no unlawful entries recorded on the eOrygen platform during the study, there were no serious adverse events experienced by participants, there was no worsening of clinical and social outcome measures, and 96% (24/25) of the young people stated that they felt safe using the platform (Multimedia Appendix 2).

In terms of symptom monitoring, the mean score for BPD symptomatology for HYPE Clinic participants reduced from 2.65 (SD 0.75) at baseline to 1.99 (SD 0.83) after the intervention. For the assessment of psychotic symptoms in EPPIC participants, the mean score for suspiciousness reduced from 2.72 (SD 1.64) at baseline to 0.94 (SD 3.78) after the intervention. In addition, the mean score for hallucinations reduced from 2.89 (SD 1.97) at baseline to 1.22 (SD 4.11) after the intervention, and the mean score for unusual thought content reduced from 1.72 (SD 1.13) at baseline to 0.67 (SD 3.74) after the intervention.

### Potential Clinical Effects

Significant pre- to postintervention improvements were observed for 9 (82%) of the 11 clinical outcome measures, including social and occupational functioning, depression, psychological distress, social anxiety, social isolation, stress, borderline symptoms, and loneliness, as well as all aspects of therapist-rated working alliance, including goal, task, and bond, with effect sizes ranging from 0.56 to 0.89 (Table 2).

**Table 2:** Inferential statistics exploring the differences between before and after the intervention in well-being and mental health symptomology.

			Participants, n		Before the intervention, mean (SD)		After the intervention, mean (SD)		Mean difference (95% CI)		Repeated measures *t* test (*df*)		*P* value		Cohen *d*
Autonomy (BPNS^a^ Questionnaire)			26		4.38 (1.08)		4.36 (1.02)		0.02 (−0.40 to 0.46)		0.13 (25)		.90		0.03
Competence (BPNS Questionnaire)			25		3.70 (0.89)		3.95 (1.01)		−0.25 (−0.64 to 0.15)		−1.30 (24)		.21		−0.26
Relatedness (BPNS Questionnaire)			26		4.44 (1.18)		4.62 (1.01)		−0.18 (−0.54 to 0.17)		−1.05 (25)		.30		−0.21
Depression (PHQ-9^b^)			25		15.92 (7.57)		13.44 (7.93)		2.48 (0.82 to 4.14)		3.08 (24)		.005		0.62
Loneliness (University of California, Los Angeles Loneliness Scale)			25		54.92 (10.54)		50.38 (10.55)		4.54 (0.75 to 8.32)		2.47 (24)		.02		0.50
Psychological well-being (Flourishing Scale)			24		33.62 (10.04)		36.79 (9.63)		−3.17 (−6.51 to 0.16)		−1.97 (23)		.06		−0.40
Social isolation (Friendship Scale)			24		13.08 (5.71)		9.83 (5.51)		3.25 (1.19 to 5.30)		3.28 (23)		.003		0.70
Social anxiety (SIAS^c^)			24		44.55 (14.43)		39.07 (15.28)		5.48 (2.34 to 8.62)		3.61 (23)		.001		0.74
Stress (PSS^d^)			24		25.33 (6.89)		19.92 (6.43)		5.42 (3.01 to 7.83)		4.65 (23)		<.001		0.95
Psychological distress (K10^e^)			24		33.24 (9.82)		29.30 (9.31)		3.94 (1.09 to 6.80)		2.86 (23)		.009		0.58
**Client working alliance**															
	Goal (WAI-SRC^f^)	24		9.63 (2.12)		9.67 (1.61)		−0.04 (−1.11 to 1.03)		−0.08 (23)		.94		−0.02	
	Task (WAI-SRC)	24		11.83 (1.17)		11.83 (1.46)		0.00 (−0.63 to 0.63)		0.00 (23)		.99		0.00	
	Bond (WAI-SRC)	23		12.39 (2.73)		12.78 (2.95)		−0.39 (−1.59 to 0.81)		−0.68 (22)		.51		−0.14	
**Therapist working alliance**															
	Goal (WAI-SRT^g^)	17		10.76 (2.08)		11.88 (2.45)		−1.11 (−1.86 to −0.37)		−3.17 (16)		.006		−0.77	
	Task (WAI-SRT)	17		10.00 (1.66)		11.00 (2.09)		−1.00 (−1.93 to −0.07)		−2.29 (16)		.04		−0.56	
	Bond (WAI-SRT)	17		16.65 (2.55)		17.53 (2.03)		−0.88 (−1.58 to −0.18)		−2.67 (16)		.002		−0.65	
Functioning (SOFAS^h^)			18		57.50 (14.63)		64.28 (17.22)		−6.78 (−10.71 to −2.85)		−3.92 (17)		<.001		−0.86

^a^BPNS: Basic Psychological Needs Satisfaction.

^b^PHQ-9: Patient Health Questionnaire (9-item version).

^c^SIAS: Social Interaction Anxiety Scale.

^d^PSS: Perceived Stress Scale.

^e^K10: Kessler Psychological Distress Scale (10-item version).

^f^WAI-SRC: Working Alliance Inventory–Short Revised (client version).

^g^WAI-SRT: Working Alliance Inventory–Short Revised (therapist version).

^h^SOFAS: Social and Occupational Functioning Assessment Scale (clinician rated).

### Young People’s Satisfaction Survey Feedback

In terms of client feedback, 88% (21/24) of the young people reported that they would use eOrygen again. The top initial reasons of interest in using eOrygen included (1) practicing well-being skills (22/24, 92%), (2) contributing to research (22/24, 92%), (3) receiving support from clinicians (17/24, 71%), (4) connecting with others with similar mental health experiences (17/24, 71%), and (5) learning and well-being (17/24, 71%). Multimedia Appendix 1 presents a full list of reasons for the young people’s use of eOrygen.

In terms of the young people’s satisfaction with eOrygen, 96% (23/24) rated it as a positive experience, 88% (22/25) rated it as easy to use, and 83% (20/25) rated it as helpful (Multimedia Appendix 2).

### Engagement

A total of 30 young people were onboarded to eOrygen (n=15, 50% from the HYPE Clinic and n=15, 50% from EPPIC). The mean *number of active days* on eOrygen was 8.4 (SD 7.5) days; 50% (15/30) of the young people had between 6 and 30 *active days*, 43% (13/30) had between 2 and 5 *active days*, and 7% (2/30) had 1 *active day* (Table 3). Of the 30 young people, 12 (40%) used the platform at least once per fortnight during the initial 6 weeks of the intervention, and 6 (20%) maintained fortnightly access across the entire 12 weeks.

In terms of therapy engagement specifically, 40% (12/30) of the young people viewed ≥3 therapy activities, 23% (7/30) viewed 1 to 2 therapy activities, and 37% (11/30) did not view any therapy activities. Half of the onboarded young people (15/30, 50%) began a therapy journey, whereas 40% (12/30) of the participants completed at least 1 journey component (ie, therapy activity within a therapy journey), 20% (6/30) completed 2 to 7 journey components, and 20% (6/30) completed ≥10 journey components (refer to Table 3 for a summary of all therapy engagement metrics).

In terms of social network engagement, it was mandatory for the young people to make an introductory post to the community as part of the onboarding process. However, almost half (14/30, 47%) of the young people made at least 1 additional post or comment, whereas 10% (3/30) of them made 2 additional posts or comments, 20% (6/30) of them made 3 to 5 additional posts or comments, and 17% (5/30) of them made >6 additional posts or comments. Furthermore, 47% (14/30) of the young people reacted to at least 1 post or comment on the social network, whereas 27% (8/30) of them reacted to >1 post or comment (refer to Table 3 for a summary of all therapy engagement metrics).

In relation to exchanging private messages with eOrygen staff using the chat function, 47% (14/30) of the young people sent at least 1 message, whereas 13% (4/30) of them sent 1 to 2 messages, 13% (4/30) of them sent 3 to 6 messages, and 20% (6/30) of them sent ≥10 messages. By contrast, 97% (29/30) of the young people received at least 1 private chat message from a clinician or peer worker, whereas 37% (11/30) of them received 1 to 3 messages, 37% (11/30) of them received 4 to 9 messages, and 23% (7/30) of them received ≥14 messages (refer to Table 3 for a summary of these metrics).

**Table 3:** User engagement with eOrygen.

Variables, n	All sites (n=30), mean (SD; range)	EPPIC^a^ (n=15), mean (SD; range)	HYPE^b^ Clinic (n=15), mean (SD; range)
Active days	8.4 (7.5; 1-30)	10.4 (8.6; 1-30)	6.3 (5.7; 1-23)
Therapy views	9.1 (21.5; 0-115)	12.9 (29.2; 0-115)	5.3 (8.5; 0-29)
Journey components completed	6.0 (18.3; 0-100)	9.3 (25.4; 0-100)	2.7 (5.2; 0-18)
Posts and comments made	3.3 (4.3; 1-22)	4.1 (5.4; 1-22)	2.6 (2.9; 1-11)
Reactions made	2.2 (4.3; 0-16)	3.3 (5.1; 0-16)	1.1 (3.1; 0-12)
Messages sent	4.6 (11.6; 0-62)	8.0 (15.8; 0-62)	1.1 (1.8; 0-6)
Messages received	8.1 (11.3; 0-60)	11.1 (15.3; 0-60)	5.2 (3.6; 1-15)

^a^EPPIC: Early Psychosis Prevention and Intervention Centre.

^b^HYPE: Helping Young People Early.

### Qualitative Findings

#### Young People

A thematic analysis of the interviews with the young people was conducted, regarding their experiences with eOrygen, with the aim of identifying facilitators and barriers to their engagement with the platform (Table 4). Facilitators included clinician endorsement, which increased trust in the platform; the presence of peer support workers, fostering a sense of safety and support; a sense of community and connection with other users; personalized therapy content recommended by clinicians; the use of eOrygen for between-sessions work, supported by follow-up discussions; and an easy-to-use interface. Conversely, barriers to engagement included general low motivation, social anxiety hindering social interactions, privacy concerns, inflexible progression in modules, and periods of limited content and interactions on the platform. Addressing these barriers and leveraging the facilitators can enhance the platform’s appeal and effectiveness for a broader range of users seeking mental health support.

**Table 4:** Facilitators and barriers to young people’s engagement with eOrygen as a blended model of care integrated into specialized services.

		Description
**Facilitators**		
	Clinician endorsement	Clinician endorsement of eOrygen seemed to increase young people’s trust in the platform. Several participants mentioned that their clinician’s recommendation to use eOrygen was central to their signing up to use the platform.
	Professional peer support workers	The presence of professional peer support workers on the platform was engaging and increased young people’s sense of safety in the community space.
	Community, connection, and belonging	Participants appreciated interacting with other young people or sharing space on the platform, even if they did not post much themselves. The sense of community and connection was a facilitator for engagement.
	Personalized therapy content	Young people found it helpful when clinicians recommended specific content aligned with their therapeutic work in face-to-face sessions. Therapeutic modules and resources within the platform were seen as valuable, trustworthy, and informative.
	Blended care	Young people commonly reported that eOrygen was used for between-sessions work. Follow-up discussions in sessions regarding the *homework* were described as helpful and valuable for consolidating learning and providing accountability to support homework completion.
	Ease of use	When participants found the platform easy to navigate and understand, it positively impacted their engagement.
**Barriers**		
	Low motivation	Several participants mentioned a general lack of motivation as a barrier to engagement.
	Social anxiety	Some participants experienced anxiety or insecurity related to social interactions on the platform. This social anxiety acted as a barrier, and participants reported feeling too anxious to post or reply to other young people’s posts. Several young people felt anxiety about the perceived lack of clarity regarding the rules of the online community.
	Privacy concerns	Privacy and confidentiality concerns were mentioned by some participants, impacting their willingness to engage fully with the platform. Some young people reported that uncertainty about who could see their posts or information raised anxiety. Several young people expressed discomfort at the thought of their face-to-face clinician viewing personal content that they may post on the platform’s social network.
	Inflexible module progression	One interviewee identified the inability to skip sections or activities within a therapy module as a barrier and requested more autonomy when engaging with therapy modules. Some participants described desiring more flexibility with how they engaged with content, such as wanting to change therapy journeys (eg, from depression to anxiety) but not being able to.
	Limited content and interactions	Periods of low activity or limited content being posted by other young people on the platform could lead to reduced engagement. Participants mentioned that the lack of new posts or interactions could be discouraging. More young people and greater interaction was requested by several young people.

#### Clinicians

The analysis of the clinician focus group grounded in the CFIR identified various barriers and facilitators to the successful implementation and use of the eOrygen intervention (Table 5). Among the notable barriers were the length of the onboarding process for young people, a need for increased confidence in using the platform, and a perceived lack of practical knowledge regarding its features and how these features are related to benefits for young people; in addition, competing priorities, such as addressing risk and acute presentations amid understaffing, consistently disrupted platform use. Conversely, the platform’s positive reputation and alignment with evidence-based frameworks emerged as facilitators. In addition, the ability to access trustworthy content was highlighted as advantageous for clinicians. These findings underscore the multifaceted nature of implementing digital mental health resources and the importance of addressing both barriers and facilitators to optimize their effectiveness.

**Table 5:** Summary of factors impacting clinician implementation of eOrygen using the Consolidated Framework for Implementation Research (CFIR).

CFIR domain	CFIR construct	Description	Illustrative quote	Implementation impact
Characteristics of individuals	Self-efficacy	Clinicians expressed varying levels of confidence when it came to implementing the intervention. This variance in confidence had a notable influence on their motivation and capacity to use the platform as part of their clinical practice.	“[H]ard when I didn’t know exactly what I was prompting them to do sometimes.” (Focus group 1)	Barrier
Inner setting	Implementation climate: relative priority	Clinicians frequently cited competing priorities, including concerns related to young people’s risk, handling acute presentations, and the challenge of being understaffed, as significant barriers that impeded their ability to engage with, and become familiar with, the eOrygen platform.	“Might’ve been a little bit me as well of not feeling like I had the time or just forgetting sometimes to just the other priorities I have.” (Focus group 3)	Barrier
Outer setting	Young people’s needs and resources	Clinicians demonstrated a strong understanding of young people’s needs, including the complexities associated with addressing these needs effectively. This encompassed underaddressed comorbid conditions, clinical complexity, and barriers related to motivation and the specific age group within the cohort.	“All the comorbidities...we don’t always get to do the, you know, anxiety management stuff or like skills-based stuff as much as, um, I feel like as much as I did in other clinics.” (Focus group 3)	Facilitator
Intervention characteristics	Complexity	Clinicians considered the process for connecting and onboarding young people to the eOrygen platform to be lengthier and more in-depth than what young people typically expect when engaging with technology.	“’Cause young people want, unfortunately, they want everything now and right there and then. So, if they click and it doesn’t work, they’ll be like, ‘now there’s nothing, it’s not working.’” (Focus group 3)	Barrier
Characteristics of individuals	Knowledge and beliefs about intervention	Clinicians had positive regard for the eOrygen platform overall. However, their implementation was hindered by a lack of comprehensive knowledge about how the platform practically worked.	“We’re all probably a bit limited in thinking about how we’re using it.” (Focus group 2)	Facilitator and barrier
Intervention characteristics	Evidence strength and quality	Clinicians had the perception that the intervention had a strong evidence base and provided quality information to support young people. However, they also had a limited understanding of which components of the intervention led to effective outcomes.	“I didn’t have to go and do my own homework or ensure that I was using like, up-to-date and relevant information.” (Focus group 3)	Facilitator and barrier

## Discussion

### Principal Findings

To the best of our knowledge, this was the first study to test an integrated blended model of care for youth psychosis and BPD in young people aged 15 to 25 years. The results of this study showed that eOrygen was feasible, acceptable, and safe. In terms of feasibility, we anticipated recruiting approximately 25 clinicians and 1 to 2 young people per clinician to the intervention and expected that the refusal rate would be <50%. Our refusal rate was 25% (15/59), which indicated 1 element of feasibility. We sought to recruit 25 clinicians; the final number of clinicians enrolled in the study was 18 (78%). In addition, we recruited 33 young people (which exceeded our goal) to the study over a 4-month period. Although we did not meet our clinician goal, our recruitment took place at the beginning of the COVID-19 pandemic, which proved a difficult period to introduce a new digital intervention to YMH services and to train clinicians in the use of a new digital platform. Despite these challenges, we still recruited a relatively high percentage of clinicians to the study and exceeded our goal in recruiting young people.

In terms of acceptability, 92% (22/24) of the young people onboarded reported that they would recommend eOrygen to others, exceeding our goal of 90%. Furthermore, 40% (12/30) of the participants used the platform at least once per fortnight during the initial 6 weeks of the intervention period, although only 20% (6/30) maintained fortnightly access across the entire 12 weeks. These findings compare well with another study reporting decreased engagement over time, with retention rates of only 3.9% over 15 days and 3.3% over 30 days for the use of mental health apps in the general population [84]. Although engagement was strong during the first 6 weeks, more strategies are needed to sustain engagement over longer periods. We did not use any strategies in this pilot to promote young people’s or clinicians’ engagement and left this to the discretion of the participating clinicians because it was purely an ecological study. Therefore, future studies should implement scalable strategies to sustain engagement for clinicians and young people, such as external support, coaching, and automated prompts or reminders.

Higher engagement rates with eOrygen were also observed for young people attending EPPIC (mean 10.4, SD 8.6 active days) versus those attending the HYPE Clinic (mean 6.3, SD 5.7 active days). To the best of our knowledge, this was the first blended model tested for young people with BPD. The MOST model was originally developed and optimized for young people with first-episode psychosis [57,60,85]. Therefore, lower engagement rates for young people attending the HYPE Clinic could be because the therapeutic model used by clinicians in face-to-face care was slightly different than the content in eOrygen, and the MOST model may need further refinement and optimization for young people with BPD. Research has indicated that young people with BPD are difficult to engage in face-to-face treatment [86-88], and it is possible that this extends to digital mental health care.

In terms of safety, there were no unlawful entries recorded on the web-based platform, no serious adverse events were experienced by participants, and there was no worsening of clinical or social outcome measures during the intervention. We also anticipated that at least 95% of young people would report feeling safe using the platform, and this goal was exceeded with 96% of the participants reporting feeling safe. Our primary findings are also in line with a previous pilot study testing Orygen Digital’s MOST platform with real-time clinician-delivered web chat counseling, which found that all acceptability and safety indicators exceeded their a priori established criteria [59].

The secondary outcome variables showed significant pre-post improvements in 9 (75%) of the 12 outcomes assessed. These included borderline symptoms, depression, loneliness, social isolation, social anxiety, stress, psychological distress, social and occupational functioning, and the therapist-reported working alliance. It is important to note that although our study included 2 clinical sites treating young people with complex mental health disorders (eg, psychotic disorders and BPD), there were no significant differences between the sites on outcome variables at baseline. Therefore, improvements in clinical outcomes relate to all participants in the study. The findings also support a previous pilot study (MOST+) that integrated MOST with real-time clinician-delivered web chat counseling [59]. MOST+ also found statistically significant improvements in psychological distress, depression, and stress. However, both studies are single-group pilot studies, and we cannot make causal inferences from the findings because it cannot be determined from uncontrolled studies whether the observed effects are related to the intervention or to external factors such as individual or in-person treatment characteristics. Future research should confirm these findings by conducting controlled studies with larger sample sizes and greater power.

Findings from a recent qualitative study also suggested that blended care has the potential to enhance the therapeutic relationship [40]. The study suggested that the TA developed through blended care can enhance engagement with both face-to-face and web-based treatment modalities by offering treatment continuity and personalization as well as enhancing therapeutic intensity, which are key areas of concern in the field [24,40]. In our study, we observed statistically significant improvements for therapist-reported TA but no improvements in client-reported TA. Research has also indicated that TA has moderate but reliable correlations with mental health outcomes [89-91]. Although we observed improvements in therapist-rated TA, they did not correlate with improvements in clinical outcomes; therefore, future research should further explore this, along with the importance of client-rated TA in relation to outcomes.

By contrast, qualitative feedback from participants in our study indicated that eOrygen was beneficial when used in a blended way; for example, young people found it helpful when their clinician recommended content to them that aligned with their in-session work, and they also found the homework to be completed on the eOrygen platform to be helpful for between-sessions work. These findings are in line with other research indicating that blended care is beneficial when it is integrated and intensifies treatment [92]. However, research has indicated that blended care may not be effective if perceived as burdensome or time consuming by clinicians, but this may be related to trial-related factors, such as reporting to a research team if using the intervention during a clinical trial or inflexible intervention structure [93]. One way to overcome this could be for developers to work with clinicians to ensure suitable content and for features to be provided within the intervention, which would enable clinicians to use the intervention with young people in a way that is meaningful, relevant, and related to the face-to-face treatment they provide [94].

### Limitations

A number of limitations should be noted. The single-group design was chosen to enhance external validity by maximizing real-world uptake of the intervention. Therefore, it was important to expose as many young people and clinicians to the intervention as possible to determine real-world uptake with eOrygen, and the inclusion of a control group may have negatively impacted the number of clinicians and young people who signed up to the intervention, potentially limiting our understanding of feasibility in this context. However, this study design limited our ability to determine a cause-and-effect relationship between the intervention and outcomes [95]. Furthermore, a 3-month time frame was chosen because this is an acceptable time frame that is comparable to the time frames of other pilot studies testing the feasibility, safety, and acceptability of digital interventions [59,66,96].

Although this study tested an integrated blended model of care, we did not collect data on how young people engaged with face-to-face treatment or how participating clinicians used the eOrygen platform, and future studies should consider this when evaluating blended models of care. Furthermore, our goal to provide flexibility and autonomy to clinicians may have negatively impacted competence in the use of the platform; for example, clinicians attended a 1-day workshop and received a printed user manual and training videos on how to use the eOrygen platform. However, as noted in the clinician-identified barriers to implementation, attending 1 workshop may be inadequate to gain competence, and there was also a substantial gap in time between clinician training and the implementation of eOrygen owing to the COVID-19 pandemic. Therefore, the training may have been forgotten, and a lack of time to review training materials may have also been an issue. Future studies should consider providing ongoing clinician support in this regard, while also remaining flexible to the needs of clinicians. Furthermore, although the recruitment goal was met for this study, it must be noted that the sample of male participants in the study was small at only 21% (7/33) of the total sample, limiting the generalizability of these findings and highlighting that significant barriers may still exist for young men to access mental health treatment [97] and that difficulties may also exist in tailoring interventions to young men [98].

### Conclusions

In conclusion, our pilot study was an important first step in testing a transdiagnostic blended model of care for youth psychosis and BPD in young people aged 15 to 25 years. We found that eOrygen was feasible, acceptable, and safe; there were indications that eOrygen may improve treatment outcomes if tested in a full-powered trial; and the majority of participants and clinicians reported positive experiences of using eOrygen as a blended model of care. However, some participants misunderstood the meaning of blended care, and future research should ensure that this is clearly outlined before integrating a digital tool into clinical practice. Furthermore, some clinicians reported a lack of knowledge and confidence in their ability to implement the intervention, and future research should aim to understand the possible barriers and address them to ensure clinician competence and confidence with the intervention itself. Overall, this pilot study provides promise for integrating blended models of care into specialized services for young people with complex mental health conditions, but a full-scale trial will be needed to test the effectiveness of such an intervention. This study also reaffirms prior findings indicating that blended models of care have the potential to increase therapeutic intensity, continuity, engagement, and effectiveness. Future research needs to focus on the development of tools to integrate blended care into practice specifically [36], as well as strategies to support both clinicians and young people in continuous use of the platform.

## Appendix

Multimedia Appendix 1 Descriptive insights into the participants’ willingness to use eOrygen again, whether they would recommend it to others, and their initial reasons of interest in using eOrygen.

Multimedia Appendix 2 Young people’s satisfaction with eOrygen (n=25).

## Abbreviations

BPD: borderline personality disorder
CFIR: Consolidated Framework for Implementation Research
DSM-5: Diagnostic and Statistical Manual of Mental Disorders
EPPIC: Early Psychosis Prevention and Intervention Centre
HYPE: Helping Young People Early
MOST: moderated online social therapy
TA: therapeutic alliance
YMH: youth mental health
