# Supplementary material for: A Novel Blended Transdiagnostic Intervention (eOrygen) for Youth Psychosis and Borderline Personality Disorder: Uncontrolled Single-Group Pilot Study
Source: JMIR Ment Health. 2024 Apr 1;11:e49217. doi: 10.2196/49217 (PMC11019426; doi:10.2196/49217)
Supplement: Multimedia Appendix 1 [file mental_v11i1e49217_app1.docx]

Multimedia Appendix 1. Descriptive Insights Into the Participants’ Willingness to Use eOrygen Again, Whether They Would Recommend It to Others, and Their Initial Reasons of Interest in Using eOrygen.

|  | Total Sample  (*N* = 24) | Clinical Sites | |
| --- | --- | --- | --- |
|  |  | HYPE  (*n* = 10) | EPPIC (*n* = 14) |
|  | *N* (% Yes) | *n* (% Yes) | *n* (% Yes) |
|  |  |  |  |
| Willingness to use eOrygen again | 21 (87.5) | 9 (90.0) | 12 (85.7) |
| Recommending eOrygen to others | 22 (91.7) | 10 (100.0) | 12 (85.7) |
| **Initial reasons of interest in using eOrygen:** |  |  |  |
| 1. Learning and well-being | 17 (70.8) | 7 (70.0) | 10 (71.4) |
| 1. Practising well-being skills (e.g., mindfulness) | 22 (91.7) | 9 (90.0) | 13 (92.9) |
| 1. Getting support from clinicians | 17 (70.8) | 6 (60.0) | 11 (78.6) |
| 1. Having increased access to support from my case manager/therapist | 16 (66.7) | 6 (60.0) | 10 (71.4) |
| 1. Chatting to other young people online | 14 (58.3) | 5 (50.0) | 9 (64.3) |
| 1. Getting support from other young people | 14 (58.3) | 6 (60.0) | 8 (57.1) |
| 1. Giving support to other young people | 11 (45.8) | 5 (50.0) | 6 (42.9) |
| 1. Connecting with others with similar mental health experiences | 17 (70.8) | 6 (60.0) | 11 (78.6) |
| 1. Relieving boredom | 11 (45.8) | 4 (40.0) | 7 (50.0) |
| 1. Relieving loneliness | 8 (33.3) | 3 (30.0) | 5 (35.7) |
| 1. Contributing to research | 22 (91.7) | 10 (100.0) | 12 (85.7) |
