# Supplementary material for: A Novel Blended Transdiagnostic Intervention (eOrygen) for Youth Psychosis and Borderline Personality Disorder: Uncontrolled Single-Group Pilot Study
Source: JMIR Ment Health. 2024 Apr 1;11:e49217. doi: 10.2196/49217 (PMC11019426; doi:10.2196/49217)
Supplement: Multimedia Appendix 2 [file mental_v11i1e49217_app2.docx]

Multimedia Appendix 2. Young People’s Satisfaction with eOrygen (N=25).

|  | Mean (SD) | Median | Values, N (%)^a^ |
| --- | --- | --- | --- |
| Positive Experience^b^ | 4.17 (0.87) | 4 | 23 (95.8) |
| Helpful^c^ | 3.67 (1.09) | 4 | 20 (83.3) |
| Easy to Use^d^ | 4.12 (1.20) | 5 | 22 (88.0) |
| Safe^e^ | 4.52 (0.77) | 5 | 24 (96.0) |
| Feeling in Control^f^ | 2.72 (1.14) | 3 | 16 (64.0) |

^a^Number of cases responding in the positive range (3 or higher) based on complete responses.

^b^Items rated from 1=not at all positive to 5=very positive.

^c^Items rated from 1=not at all helpful to 5=very helpful.

^d^Items rated from 1=not at all easy to 5=very easy.

^e^Items rated from 1=not at all safe to 5=very safe.

^f^Items rated from 1=not at all in control to 5=very much in control.
